# The patient perspective on overactive bladder: a mixed-methods needs assessment

**DOI:** 10.1186/1471-2296-15-96

**Published:** 2014-05-14

**Authors:** Frank A Filipetto, Kimberly G Fulda, Amy E Holthusen, Thomas M McKeithen, Pam McFadden

**Affiliations:** 1 Department of Family Medicine, University of North Texas Health Science Center, 855 Montgomery Street, Fort Worth 76107, USA; 2 Texas Prevention Institute, University of North Texas Health Science Center, (3500 Camp Bowie Blvd.), Fort Worth 76107, USA; 3 Interstate Postgraduate Medical Association, P.O. Box 5474 Madison 53705, USA; 4 Healthcare Performance Consulting, Inc. (2321 Stockton Drive), Fleming Island 32003, USA; 5 Department of Professional and Continuing Education, University of North Texas Health Science, Center (3500 Camp Bowie Blvd.), Fort Worth (76107), USA

**Keywords:** Overactive bladder, OAB, Needs assessment, Patient survey, Patient perspective

## Abstract

**Background:**

While overactive bladder is often managed in the primary care setting, a number of barriers including embarrassment, poor communication, and low patient adherence contribute to the under-treatment of patients with burdensome urinary symptoms. In order to address these challenges, it is crucial to have a fundamental understanding of patient perspectives toward OAB and urinary symptoms. To meet this aim, researchers designed and conducted a study to identify patients’ knowledge, experiences and attitudes, barriers to treatment adherence, and desires and tendencies regarding patient/clinician communications.

**Methods:**

A mixed-methods qualitative/quantitative needs assessment of patients with overactive bladder and/or urinary symptoms. Researchers conducted in-depth qualitative interviews via telephone with 40 patients. Interview results informed the design and dissemination of a quantitative survey, which was completed by 200 self-selected respondents who had previously identified themselves as having overactive bladder or bladder problems. Statistical and qualitative analysis of results were conducted.

**Results:**

Among survey respondents, an average of 3.5 years elapsed between symptom onset and seeking diagnosis by a physician. In the long term most patients do not experience improvement in symptoms. Medication non-adherence is common and is related to therapy effectiveness and adverse effects. Patients clearly indicate that communication and patient/physician relationships are important to them and they would prefer the clinician initiate the conversation on overactive bladder. Patient experiences, perspectives, and attitudes toward their bladder symptoms differ in many ways from clinicians’ assumptions.

**Conclusions:**

The significant time gap between symptom onset and diagnosis indicates ongoing need for screening and diagnosis of overactive bladder. Contrary to guideline recommendations, urinalysis and physical examination are not widely used in clinical practice. Many patients experience no improvement in symptoms over time. Patients indicate that clinician/patient relationships and communication regarding their condition are important.

## Background

Overactive bladder (OAB) is a clinical symptom complex that affects a significant number of primary care patients in the United States. It is defined by the International Continence Society (ICS) as the presence of urinary urgency, usually accompanied by frequency and nocturia, with or without urgency urinary incontinence, in the absence of urinary tract infection or other obvious pathology [1]. The estimated worldwide prevalence of OAB is anticipated to increase from 10.7% in 2008 to 10.9% by 2018, affecting 546 million individuals [2]. A recent study suggests that only 45.7% of patients with probable OAB discussed the symptoms with a health care provider and only 8.1% were receiving treatment [3]. While usually not life threatening, symptoms of OAB significantly lower quality of life by adversely affecting self-esteem, family relations, sexual satisfaction, professional and social life, and overall perception of health [4-7]. In addition, the direct financial costs related to diagnosis, treatment, and consequences of the disease were estimated at $12.6 billion during 2000 [8].

While OAB can often be diagnosed and managed successfully in the primary care setting, several barriers contribute to the under-treatment of patients with these burdensome symptoms. Primary care providers may lack training in this area or lack clinical awareness of effective evaluation and management strategies. One survey of general practitioners found the need for increased awareness among clinicians and the general population. Inadequate communication between patients and providers has limited successful diagnosis, treatment, and management of OAB [9,10]. Many patients are embarrassed or reluctant to mention urinary symptoms during office visits. Patients may feel their symptoms are not severe enough to warrant consulting a clinician, do not represent a valid medical condition, or perceive their symptoms to be unavoidable outcomes of aging or comorbidities [9]. For example, a recent survey found that more than half of women who discussed OAB with a provider had waited more than a year to seek treatment for their symptoms [9] and only 44% of men with symptoms of urinary incontinence had consulted a physician [11].

Another barrier to effective management of OAB is poor patient adherence or persistence to treatment [12]. The causes of non-adherence are multifactorial and may include unclear or unrealistic treatment goals, side effects or inconvenience of therapy, cost, or simply forgetting to follow a treatment regimen.

The clinical needs in the diagnosis, management, and treatment of OAB are well established. Current evidence-based medical literature documents the scope of the problem, its effect on patient health and quality of life, and guidelines for effective diagnosis and management. Successful clinical outcomes and patient satisfaction are dependent upon timely identification of symptoms, appropriate evaluation, effective communication and patient education, and realistic treatment plans and goals. To improve clinical outcomes and patient satisfaction, it is essential that health care providers have a fundamental understanding of patient perspectives toward OAB and incontinence. Prior studies reflecting patient perspectives have identified the negative effects of OAB on health-related quality of life [13] as well as dissatisfaction with OAB care and miscommunication [14]. With the goal of improving the quality of care for patients with OAB, the intent of this study was to contribute additional patient perspective related to knowledge and attitudes, symptoms and history, screening, testing and diagnosis, management, satisfaction of treatment, adherence to treatment, and provider/patient communication. Results will provide needed complementary patient perspective to existing data on clinician practice gaps regarding treatment and management of OAB and incontinence.

## Methods

This IRB-approved study consisted of qualitative in-depth patient interviews and quantitative online patient surveys. Survey respondents consisted of individuals in the ResearchNow database that had previously self-identified as having symptoms of OAB or bladder dysfunction. ResearchNow is a company that provides researchers access to panels of individuals related to various topics. ResearchNow recruited participants for the interviews and survey based on eligibility criteria. Researchers employed a double opt-in process in which participants who opted in after receiving a personalized e-mail invitation were sent a follow up e-mail confirmation validating their participation a second time. The protocol was approved by the University of North Texas Health Science Center Institutional Review Board.

### Qualitative interviews

Forty in-depth qualitative interviews were conducted with patients who were pre-screened for a history of OAB and/or urinary incontinence. Interviews lasted 30–60 minutes and were conducted by study-specific trained telephone interviewers. The purpose of the interviews was to identify issues and influences on clinical behavior from a patient perspective. These interviews also helped to identify key areas where further clinician/patient educational interventions may be needed and provided the basis for development of the patient survey. Questions pertaining to the type of treatment and adherence to treatment for OAB were open ended in the qualitative interviews. Verbal consent was obtained from participants via telephone before the interviews were conducted.

### Survey

A quantitative patient survey and assessment tool (Additional file 1) was developed based on responses from the qualitative interviews and distributed via e-mail to individuals who self-identified as experiencing OAB and/or urinary incontinence. Participants were eligible if they had experienced symptoms of urgency, frequency, leakage, and/or urinary incontinence. A total of 200 surveys were completed. The survey took fewer than 15 minutes to complete on average and consisted of two components focusing on patient experience and knowledge/attitudes. The experience portion of the assessment gathered information about actual patient and clinician behaviors pertaining to the assessment, diagnosis, and management of OAB, including patient perceptions about how OAB had been managed by their personal healthcare provider. The patient knowledge and attitude portion of the assessment quantified patients’ basic knowledge of the epidemiology, pathophysiology, diagnosis, and treatment of OAB. This tool also assessed attitudes about OAB, its treatments, and providers who manage this condition. The following treatments for OAB were assessed in the survey: medication, pelvic floor muscle exercises, reducing liquids, reducing caffeine, bladder training, changes in diet, and botox injections into the bladder. Subjects were also given an “other” option to discuss any treatments not covered in the list. A cover letter that explained confidentiality issues and other consent elements was included with each survey.

### Data analysis

Data analyses for survey responses were conducted using SPSS. Descriptive statistics such as means and frequencies are provided. Differences in knowledge scores for respondent gender, respondent age (41–60 and ≥ 61 years), physician gender, and physician specialty were determined using analysis of covariance (ANCOVA) to control for the lag time between when the respondent first noticed symptoms and when he/she talked with a doctor about their symptoms. Analysis of variance (ANOVA) was used to determine the difference in knowledge score with frequency of communication between the patient and physician and type of educational material utilized. Participants were asked if they agree or disagree with the statement “OAB is just a part of aging, I just have to live with it”. Responses were scored on a Likert scale, and the mean score was compared between age group and gender using an independent sample t test. All included surveys had complete data.

## Results

Interviewees included 31 females and 9 males ranging from 40 to 80 years of age, with an average age of 63. They had a 3–15-year history of bladder and/or urinary symptoms. Most of the interviewees had a primary care provider to manage their symptoms. Several men were treated by urologists, and two women were cared for by urogynecologists. Two hundred U.S. participants from 37 states completed the quantitative survey assessment. Responses from six participants were found to be incomplete and were excluded from analysis, resulting in a sample size of 194. Ninety-eight (51%) were men and 96 (49%) were women. Sixty-four (33%) participants were 41–60 years of age, and 130 (67%) of participants were 61 years of age or older.

### Patient knowledge and attitudes

Interviewees demonstrated a good understanding of their own condition and treatments. There was mixed understanding of treatment methods and goals of therapy in general. Patient knowledge and attitudes were assessed via survey based on correct responses to 6 questions. Participants overall demonstrated a fairly high level of knowledge about OAB, with a 65% correct response rate on average. Both female respondents (p < 0.001) and respondents who saw a female physician (p < 0.001) scored higher, on average, on the knowledge scale as compared to male respondents and respondents who saw a male physician. Additionally, respondents who saw urogynecologists or obstetrician/gynecologists had higher average scores as compared those who saw a primary care physician, urologist, or other physician (p = 0.004). Patient age, frequency of communication, or type of educational material provided had no statistical association with overall patient knowledge of OAB. Regarding attitudes about OAB, the majority of respondents (65.5%) disagreed with the statement ‘OAB is just part of aging; I just have to live with it’. Further analysis reveals that while there was no difference in response to this question according to age group, men were statistically more likely to agree with the statement (p < 0.033).

### Symptoms and history

Interviewees reported having experienced common symptoms of OAB such as urgency, frequency, nocturia, and leakage. Many had little to no urinary incontinence beyond minor leakage, yet they reported that symptoms were troublesome and that the effect on daily life was significant. Participants reported taking a number of deliberate steps to self-manage their symptoms such as using pads and restricting fluids. Several reported bathroom mapping and restricting their travel to short trips. Quantitative survey results confirmed these findings with 76% of respondents reporting that they ‘try to learn where bathrooms are located so [they] can get to them quickly if needed’. Responses also showed that participants often suffered for long periods of time before consulting a clinician. On average, respondents had experienced urinary and bladder symptoms for 9 years (sd = 9.3) while only being under a physician’s care for these symptoms for 5.8 (sd = 6.0) years, resulting in an average time gap of 3.5 years between symptom onset and treatment initiation.

### Disease screening

Interviews suggested that physicians seldom initiate communication about urinary or bladder issues. Participants reported that physicians had engaged patients regarding symptoms after the participants themselves had mentioned them. Patients, however, prefer that their provider initiate discussion about urinary symptoms because they find it embarrassing to bring the topic up themselves. Survey results show similar findings in which 42% of respondents raised the issue of urinary or bladder symptoms themselves while seeing their health care provider for an unrelated concern. Only 14% of survey participants reported that ‘my doctor asked me about urinary or bladder symptoms’.

### Testing and diagnosis

Qualitative interviews assessing testing and diagnosis of OAB showed that many interviewees had a urinalysis performed during their assessment. Some reported having post-void residuals, urine flow studies, vaginal exams, or stress testing performed. Other patients had undergone no specific testing, including physical examination and urinalysis, despite current guidelines from the American Urological Association recommending that these be performed on all patients with signs and symptoms of OAB [15]. Survey results confirm that urinalysis was the most commonly performed test, though only 56% of respondents reported its use during their evaluation. Only 44% reported having had a physical examination (vagina or prostate), while 40% reported a bladder-emptying test.

### Management

Interviewees reported that they felt physicians were quick to prescribe medication even when the patients did not necessarily think it was needed. Sixty-two percent of survey respondents indicated that medications had been recommended or prescribed by their doctor. Specific pharmacologic treatments mentioned included anti-muscarinic agents, α–blockers, and vaginal estrogen cream. Few patients reported that their physician offered non-pharmacologic or behavioral management of their symptoms. Only 29% and 31% of surveyed participants reported being provided bladder training and pelvic floor exercises, respectively. Some patients indicated that they were on no treatment by their own choice, either having never started or having discontinued their medications due to side effects and/or lack of efficacy. Side effects reported as a cause of medication cessation included the common anticholinergic effects of dry mouth, constipation, and blurred vision.

When questioned about the importance of managing OAB as compared to other medical conditions, interviewees indicated that OAB symptoms bother them enough that they want to have it addressed by their physician. Similarly, survey results showed that 31% of respondents believe it is ‘very important’ that their OAB or urinary problems be treated. Only 1% indicated that it was ‘not at all important’.

### Satisfaction & effectiveness of treatment

Regarding satisfaction with current treatment regimens, most responses indicated moderate satisfaction with only 9% of survey respondents being ‘very satisfied’ with their current treatment. In order to assess the effectiveness of therapy, survey participants were asked to rate both their initial and current symptoms on a Likert scale from 1 (not bothered) to 5 (very bothered). Responses showed virtually no change in symptom severity between the time of symptom onset and present day.

### Treatment adherence

Adherence to treatment as reported by interviewees was low overall, initially starting high but ultimately falling over time. Adherence decreased as patients perceived treatments becoming less effective. Often titration to higher doses of medications occurred, sometimes resulting in increased side effects and eventual dosage reduction or discontinuation. Medication non-adherence was mostly a result of side effects, though ineffectiveness and cost were cited as other reasons. Adherence to treatment with medication was higher than adherence to lifestyle changes and pelvic floor exercises. Ineffectiveness and not remembering were the key reasons that patients failed to continue with less invasive treatments such as pelvic floor exercises. Respondents who reported more frequent communication with their provider about OAB had higher levels of adherence than those with less frequent communication (p = 0.018).

### Provider/patient communication

Interviewees reported overall dissatisfaction with clinicians’ frequency and quality of communication regarding OAB. While most felt their physicians have an understanding and appreciation of their condition, some reported that their provider ‘doesn’t get it at all’. Physician understanding was equated with being a ‘good listener’. Communication between female patients and male physicians seemed the most problematic with women reporting such impressions as ‘he doesn’t understand’ , ‘is not concerned’ , or ‘this is not important to him’.

Survey respondents reported the frequency of OAB discussions with their physicians. Forty-one percent said that they discussed urinary or bladder symptoms ‘occasionally’ , while 32% reported discussions ‘on nearly every visit’. These findings were similar to interview responses where follow-up conversations initiated by either patient or physician after initial diagnosis and treatment rarely occurred. It is evident that patients prefer regular discussion of OAB with their physician, with 75% of those surveyed rating this issue as ‘very important’.

### Patient education

Interview responses to questions about patient education were variable. Patients reported receiving mixed explanations with varied terminology from clinicians. Some patients utilized the terminology ‘overactive bladder’ or ‘OAB’. Others mentioned ‘incontinence’ or ‘urge incontinence’, but when questioned further, described the symptoms of OAB. The amount of detailed information that was provided to patients was mixed; some patients received little to no information about OAB while others received educational materials in the form of printed brochures. Many patients had conducted their own research regarding OAB via the internet. Survey results were similar; 57% of respondents reported receiving no educational materials and 37% reported receiving printed information.

## Discussion and conclusions

This study provides clinicians a much-needed patient perspective regarding the diagnosis, treatment, and management of OAB that can be utilized to improve patient-centered outcomes. It is evident that there exists a need for improved screening and diagnosis of OAB. This study identified a significant time period (3.5 years) between onset of symptoms and eventual diagnosis by a clinician. In addition, only a small minority of clinicians questioned patients about urinary symptoms. Though it was more common for patients to initiate the discussion about bladder issues, the embarrassment and reluctance to discuss the topic presents a major barrier for patients, ultimately resulting in unidentified and untreated symptoms. Proactive screening and communication on the part of the clinician via simple office strategies may make a significant difference in ensuring timely diagnosis.

Despite available published guidelines for the diagnosis and evaluation of patients with symptoms of OAB [15], results revealed that appropriate diagnostic and assessment tools were not widely used in clinical practice. Though urinalysis and physical examination should be performed on all patients with symptoms of overactive bladder, a very large percentage of the study population did not have them performed. This represents a significant practice gap for health care providers and its cause merits further investigation.

With regard to outcomes of treatment for OAB, it was clear that in this study patients’ long-term therapy needs were not being met. Patients reported virtually no change in the severity of symptoms from onset to present day, despite an average time gap of almost 9 years. This may be the result of several barriers that include unrealistic expectations, lack of provider follow-up and communication, providers not following treatment guidelines, and non-adherence to treatment due to side effects and/or cost. Despite being effective, non-invasive, and having virtually no adverse effects, behavioral therapies such as bladder training and pelvic exercises were associated with the lowest adherence rates in this study. Similarly, low adherence rates to behavioral interventions such as pelvic-floor muscle exercises and delayed voiding are low among women with urge incontinence when prescribed in adjunct to drug therapy [16]. Providers will need to identify and implement proven strategies to assess and encourage adherence by defining clear treatment goals, creating a treatment plan utilizing educational and instructional materials, and frequently communicating with patients to identify potential barriers to adherence.

Patients indicated that communication and patient/physician relationships are important. They prefer that the provider initiate conversations about bladder issues and value ongoing communication and follow up after diagnosis. In addition, patients who communicated more frequently with their providers had higher adherence to treatment. This is not surprising since physician/patient communication is associated with treatment adherence [17]. Communication should clearly define treatment goals and strategies prior to initiating drug therapy. Many patients felt providers were too quick in prescribing medications. Patients seek knowledge about their condition and often have to obtain this information from the internet, since the majority of patients in this study were not provided with any educational materials about their condition. Providers need to make an effort to identify and provide educational and training resources to patients with OAB.

Current health care models emphasize communication, clinical outcomes, and patient satisfaction. This study has helped identify patients’ experiences and perspectives related to the overall diagnosis and management of OAB. In doing so, it has identified practice gaps related to communication, timely diagnosis, evaluation, treatment, long-term follow-up, adherence to therapy, and education. Improving performance in each of these areas would serve to improve the quality of life of those patients suffering from the symptoms of OAB.

Several limitations to this study should be noted. First, the survey was developed using responses from the qualitative interviews. The survey was not pilot tested and was not validated. Therefore, the results should be interpreted with caution. Additionally, participants were recruited using a company that compiles panels of participants. This introduces a selection bias that may or may not affect the overall outcomes. The results may not represent the view of all patients with OAB. Finally, the International Continence Society’s definition of OAB was not used. The current research provides pilot data to develop a larger study to examine the patient’s perspective of OAB and their quality of life.

## Abbreviations

OAB: Overactive Bladder; ICS: International Continence Society; U.S.: United States.

## Competing interests

The authors declare that they have no competing interests.

## Authors’ contributions

FF reviewed needs assessment data and final report and wrote the manuscript. KF led IRB submission and completed statistical analysis of quantitative data. AH participated in writing the application for the original project grant, participated in data analysis, wrote the final report, and finalized the manuscript. TM led methodology design, survey design, and data collection/analysis. PM served as Principal Investigator for the project. All authors read and approved the final manuscript.

## Pre-publication history

The pre-publication history for this paper can be accessed here:

http://www.biomedcentral.com/1471-2296/15/96/prepub

## Supplementary Material

Additional file 1 **Patient Survey Instrument.** Blank copy of the survey that was administered to patients. Click here for file
