# Supplementary material for: The patient perspective on overactive bladder: a mixed-methods needs assessment
Source: BMC Fam Pract. 2014 May 14;15:96. doi: 10.1186/1471-2296-15-96 (PMC4030445; doi:10.1186/1471-2296-15-96)
Supplement: Additional file 1 — Patient Survey Instrument. Blank copy of the survey that was administered to patients. [file 1471-2296-15-96-S1.pdf]

The Patient Perspective on Overactive Bladder:
A Mixed-Methods Needs Assessment
Patient Survey Instrument

Thank you for your participation in this survey. This project is sponsored by the University of North Texas Health Science Center. The purpose of the survey is to better understand some of the problems that occur for patients with overactive bladder. Your answers are entirely confidential. Neither your name, nor any information that would personally identify you, will be provided in our report. All information from this survey will be grouped and summarized with information from other patients for our report. The survey will take no more than 10 minutes.

1. Please indicate your age:
   a. Less than 40 years old
   b. 41-60 years old
   c. 61-80 years old
   d. More than 80 years old

2. Please indicate your gender
   a. Male
   b. Female

This survey will ask you questions about problems you have had with your bladder or urinary system that may have resulted in symptoms such as frequent urination, wetting the bed, leakage, or urgent need to use the bathroom. Please answer all questions with respect to these symptoms and how they were managed by your doctor.

3. For urinary and bladder symptoms, please indicate what kind of doctor you see (select the type that sees you for these symptoms):
   a. Primary care physician (Family medicine, general internal medicine, general practice, etc.)
   b. Urologist
   c. Urogynecologist
   d. OB/GYN
   e. Not sure
   f. Other (fill-in)

4. Is this doctor a male or a female?
   a. Male
   b. Female

5. How did this problem come up in the physician's office? (select the best answer)
   a. I had an appointment for this specific reason
   b. I had an appointment for something else, but brought it up to my doctor because the symptoms were bothering me

   c. It was on a questionnaire that I filled out in the office.
   d. My doctor asked me about urinary or bladder symptoms when I was seeing him for another problem, or routine checkup
   e. I don't remember
   f. Other (fill-in)

6. How do you think your doctor would rate the importance of your urinary or bladder problems, as compared to the rest of your medical conditions?

	Not at all important 1	2	3	Very Important 4
	❏	❏	❏	❏

7. How important is it to you that your primary care doctor and the specialist (urologist or urogynecologists) work together to help with your urinary or bladder problems?

	Not at all important 1	2	3	Very Important 4
	❏	❏	❏	❏

These next few questions will help us to determine your understanding of bladder and urinary problems. Please answer them as best you can!

8. Overactive bladder is much more common in women than men
   a. True
   b. False

9. The definition of overactive bladder includes: (select the best single answer)
   a. Accidently leaking urine when laughing or coughing
   b. A sudden and urgent need to urinate
   c. Leaking when exercising
   d. Painful urination

10. Overactive bladder (OAB) is caused by:
   a. Spasms of the bladder muscle
   b. An enlarged prostate gland
   c. Drinking too many fluids

11. It is normal to need to go to the bathroom:
    - **a. Less than 8 times a day**
    - b. 9-12 times a day
    - c. More than 12 times a day

12. The following are effective in reducing leakage: (check all that apply)
    - **a. Reducing liquid intake**
    - **b. reducing caffeine**
    - **c. Pelvic floor (Kegel) exercises**
    - **d. Increased exercise**
    - e. Reduced sexual activity
    - **f. Bladder training**

These next few questions deal with your personal feelings about urinary and bladder symptoms. There are no right or wrong answers.

13. How important is for your bladder or urinary problems to be treated, as compared to the rest of your medical conditions?

	Not at all important 1	2	3	Very Important 4
	❏	❏	❏	❏

14. How important is it for you to discuss OAB with your doctor on a regular basis?

	Not at all important 1	2	3	Very Important 4
	❏	❏	❏	❏

15. Rate your agreement with the following statement: "OAB is just a part of aging, I just have to live with it

	Disagree Strongly 1	2	3	Agree Strongly 4
	❏	❏	❏	❏

16. How satisfied are you with the treatments you are currently on?

	Very Dissatisfied1	2	3	Very Satisfied4
	❏	❏	❏	❏

To the best of your ability think back to when you first began to experience difficulties with urination or bladder problems.

17. How long ago (in years) did you first notice urination or bladder problems? (put 1 if 1 year or less)

18. How long ago (in years) did you first speak with a doctor about these symptoms? (put 1 if less than 1 year)

For the next set of questions, please rate each item twice. Please rate the symptoms you had when you first noticed urinary or bladder problems, and the symptoms you have now.

19. First symptoms [1=Not bothered, 4=Very bothered]

	1	2	3	4
An uncomfortable urge to urinate?	❏	❏	❏	❏
A sudden urge to urinate with little or no warning?	❏	❏	❏	❏
Accidental loss of small amounts of urine?	❏	❏	❏	❏
Urinating while asleep?	❏	❏	❏	❏
Waking up at night because you need to urinate?	❏	❏	❏	❏
Urine loss associated with a strong desire to urinate?	❏	❏	❏	❏

20. Current Symptoms [1=Not bothered, 4=Very bothered]

	1	2	3	4
An uncomfortable urge to urinate?	❏	❏	❏	❏
A sudden urge to urinate with little or no warning?	❏	❏	❏	❏
Accidental loss of small amounts of urine?	❏	❏	❏	❏
Urinating while asleep?	❏	❏	❏	❏
Waking up at night because you need to urinate?	❏	❏	❏	❏
Urine loss associated with a strong desire to urinate?	❏	❏	❏	❏

21. Please indicate how your lifestyle was changed due to urinary or bladder symptoms (check all that apply)
    a. Reduced social activities like going out to dinner or visiting friends
    b. Reduced sports activities or exercise
    c. Use pads or adult diapers
    d. Avoid long car rides or other travel
    e. Try to learn where bathrooms are located so I can get to them quickly if needed
    f. Other

22. Please list any tests you have had related to urinary or bladder symptoms.
    a. Urine test
    b. Cystoscopy
    c. Bladder-emptying test
    d. Vaginal/prostate exam
    e. None
    f. Other

23. Please check any treatments that your doctor has recommended or prescribed (all that apply):
    a. Medication to control bladder symptoms
    b. Pelvic floor muscle exercises
    c. Reducing liquids
    d. Reducing caffeine
    e. Bladder training
    f. Changes in my diet
    g. Botox injection into the bladder
    h. No treatments
    i. Other _______________________

24. These are the treatments that you said your doctor has recommended or prescribed. Please indicate next to each one how well you have been able to stick to the treatment. (Only shown are those treatments selected in Question 23)

	Not at all 1	2	3	Very well 4
Medication to control bladder symptoms	❑	❑	❑	❑
Pelvic floor muscle exercises	❑	❑	❑	❑
Reducing liquids	❑	❑	❑	❑
Reducing caffeine	❑	❑	❑	❑
Bladder training	❑	❑	❑	❑
Changes in my diet	❑	❑	❑	❑
Botox injection into the bladder	❑	❑	❑	❑
Other _______________________	❑	❑	❑	❑

25. You indicated that you found it hard to keep to the following treatments. Please indicate the main reason why it was difficult. (Only those treatments rated as a “Not at all, 1” on Question 24).

	Cost of treatment	Side effects	Inconvenient	Doesn’t work	I forget to do it	Other
Medication to control bladder symptoms	❑	❑	❑	❑	❑	❑
Pelvic floor muscle exercises	❑	❑	❑	❑	❑	❑
Reducing liquids	❑	❑	❑	❑	❑	❑
Reducing caffeine	❑	❑	❑	❑	❑	❑
Bladder training	❑	❑	❑	❑	❑	❑
Changes in my diet	❑	❑	❑	❑	❑	❑
Botox injection into the bladder	❑	❑	❑	❑	❑	❑
Other _______________________	❑	❑	❑	❑	❑	❑

26. How often do you discuss urinary or bladder symptoms with your doctor?
    a. On nearly every visit
    b. Occasionally
    c. Only once or twice when I was diagnosed
    d. Never

27. Did your healthcare provider offer educational materials, such as:
    a. Printed information sheet or brochure
    b. Video
    c. Website
    d. Other (fill-in) ____________________
